# Insights into pathogenic events of HIV-associated Kaposi sarcoma and immune reconstitution syndrome related Kaposi sarcoma

**DOI:** 10.1186/1750-9378-3-1

**Published:** 2008-01-21

**Authors:** Liviu Feller, Johan Lemmer

**Affiliations:** 1 Department of Periodontology and Oral Medicine, Box D26 School of Dentistry, University of Limpopo Medunsa Campus, Pretoria, South Africa

## Abstract

A decrease in the incidence of human immune deficiency virus-associated Kaposi sarcoma (HIV-KS) and regression of some established HIV-KS lesions is evident after the introduction of highly active anti-retroviral treatment (HAART), and is attributed to generalized immune restoration, to the reconstitution of human herpesvirus (HHV)-8 specific cellular immune responses, and to the decrease in HIV Tat protein and HHV-8 loads following HAART. However, a small subset of HIV-seropositive subjects with a low CD4+ T cell count at the time of introduction of HAART, may develop HIV-KS as immune reconstitution inflammatory syndrome (IRIS) within 8 weeks thereafter.

## Introduction

Kaposi sarcoma (KS) is the most common human immunodeficiency virus (HIV)-associated neoplasm [1]. HIV-associated Kaposi sarcoma (HIV-KS) lesions are characterized microscopically by angiogenesis, the presence of spindle-shaped tumour cells, inflammatory cell infiltrates dominated by mononuclear cells, extravasated erythrocytes and oedema [2-4]. Clinically, HIV-seropositive subjects with KS exhibit mainly multifocal mucocutaneous patches, plaques and nodules, and less frequently organ involvement [5-7]. The most common site of extra-mucocutaneous HIV-KS involvement is the lymph nodes followed by the gastro-intestinal tract and the lungs. The oral cavity is commonly affected and is the initial site of involvement with KS in about 20% of HIV-seropositive subjects with KS [8]. Oedema is a major clinical feature associated with advanced KS. It results most frequently when a local inflammatory reaction induced by production of cytokines by KS cells is complicated either by lymphatic obstruction by the enlarging tumour itself, or less commonly by proximal lymph node involvement [9]. The pathogenesis of HIV-KS is complex, and involves interaction between human herpesvirus (HHV)-8, HIV, inflammatory cytokines, and angiogenic factors in the presence of profound immune suppression [10-12]. However, the understanding of how these multiple factors interplay to initiate KS is incomplete [3].

HHV-8 is present in all four epidemiological forms of KS (classic, endemic, iatrogenic and HIV-KS). Seroprevalence studies demonstrate that HHV-8 DNA in peripheral blood mononuclear cells (PBMC) and specific antibodies to HHV-8 are associated with increased risk of KS, and there is a positive correlation between the HHV-8 viral load and the severity of KS. These lines of evidence indicate that HHV-8 is necessary for the development of KS, but since HHV-8 seroconversion in the general population is not uncommon and is much more common in HIV-seropositive subjects, but yet only some members of these populations develop KS, other co-factors are clearly necessary for the development of KS [13-29].

HIV contributes to the pathogenesis of KS through several mechanisms: HIV Tat protein directly promotes HHV-8 replication [30,31]; HIV induces the production of inflammatory cytokines [12,32], and causes a profound immune impairment that is conducive to the development of KS. The incidence and aggressiveness of KS is substantially increased in HIV-seropositive subjects compared to HIV-seronegative subjects [10,31,32]. This emphasizes the important role of HIV in the natural course of HIV-KS.

The use of highly active antiretroviral therapy (HAART) has resulted in a dramatic reduction in the morbidity and mortality in HIV-seropositive subjects [33-36]. HAART, although not directly affecting HHV-8 replication, indirectly brings about a decrease in HHV-8 viral load [37], a substantial reduction in the prevalence and incidence of HIV-KS [38-42], and improvement in the clinical manifestation of KS [43-52].

However, HAART does not ensure that KS will not develop, and in subjects receiving HAART, KS remains the most frequent HIV-associated neoplasm [5,53]. HIV-seropositive subjects who had already received HAART at the time of KS diagnosis, usually have less aggressive KS disease compared to HIV-seropositive subjects who were HAART naïve at the time of KS diagnosis [5,54]. In addition, KS sometimes recrudesces as an immune reconstitution inflammatory syndrome (IRIS) in HIV-seropositive subjects shortly after the introduction of HAART, despite an improvement in the CD4+ T cell count and controlled HIV viremia [55-60].

## The natural course of HIV-KS

There is a compelling body of information that supports the concept that HIV-KS is an opportunistic tumour that starts as a reactive hyperplasia and eventually may progress to a true neoplasia [12,13,16,23,60-64].

HIV-KS has its origin in an environment induced by inflammatory T helper (Th)-1 cytokines associated with a marked impairment of cellular immune responses, brought about by HIV infection. The inflammatory infiltrate in HIV-KS lesions comprise CD8+ T cells, monocytes, macrophages and dendritic cells. These cells produce inflammatory cytokines that together with HHV-8 gene products, activate endothelial cells and trigger the development of HIV-KS. Early HIV-KS lesions manifest clinically as indolent red-purple macules or papules, that show proliferation of endothelial cells and formation of slit-shaped vascular channels resembling well vascularized exuberant granulation tissue. In time, HIV-KS lesions become nodular and may have an aggressive clinical behaviour [60,61].

The late-stage maculo-papular HIV-KS lesions are characterized by proliferation of spindle cells, of lymphatic and/or blood vascular endothelial origin. The spindle cells become the predominant cell type, though the vascular element is always evident [23,65,66]. The progression of HIV-KS is attributed to dysregulation in cell cycle growth and resistance to apoptotic signals mediated by altered cytokine networks, latent and dysregulated lytic HHV-8 genes and HIV Tat protein [3,67].

Early-stage HIV-KS is a polyclonal reactive angioproliferative disorder. This is evident from the multifocal characteristic of the HIV-KS lesions in the absence of metastasis; by the occasional regression of HIV-KS lesions either spontaneously or following the introduction of HAART; and by the lack of clonality [68]. In contrast, late stage HIV-KS lesions from disparate subjects may show a spectrum of multiclonal origin, monoclonality, oligoclonality and polyclonality, and it is likely that a subset of late-stage lesional cells of monoclonal origin undergo malignant transformation [69,70].

Most of HIV-KS spindle cells express HHV-8 latent genes, but not genes that are involved in lytic reactivation and replication of latently infected cells. However, some of the virally infected KS cells express HHV-8 lytic genes, that provide paracrine angioproliferative inductive signals to neighbouring endothelial and spindle cells mediating angiogenesis and spindle cell proliferation [23].

The persistent endothelial and spindle cell proliferation, in response to HHV-8 induced inflammatory and growth factors, and to HHV-8 oncogenes leads to dysregulated cell proliferation and survival, followed by cellular transformation and eventual progression to a monoclonal tumour [12,13,23]. The malignant transformation of HIV-KS cells, when and if it occurs, is probably driven by HHV-8 latent oncogenes and by the dysregulated constitutive activity of viral G protein-coupled receptor (vGPCR) expressed outside the lytic phase of the virus replication cycle, without notable cellular genetic and epigenetic mutation of cell cycle genes and/or tumour suppressor genes [3,62].

## The implication of inflammatory cytokines in the pathogenesis of HIV-KS

The pathogenesis of HIV-KS is related to infection with HHV-8 and HIV, and to persistent inflammation in the presence of high level of Th1-type inflammatory cytokines including tumour necrosis factor (TNF)-α, interferon (INF)-γ, interleukin (IL)-1β, and IL-6, within an environment of immunosuppression. Increased HIV and HHV-8 loads and concurrent opportunistic infections only serve to perpetuate the inflammatory state [60,71]. It is probable that the increased level of inflammatory and angiogenic cytokines in all epidemiological forms of KS is the consequence of the local host immune activation against HHV-8 and the HHV-8 release of cytokines in response to the output of the HHV-8 genes [11,13].

High levels of inflammatory cytokines in early KS lesions trigger endothelial cells to express activation markers including vascular cell adhesion molecules, matrix metalloproteinase (MMP), α_5_β_1 _and αVβ_3 _integrins, growth factors such as vascular endothelial growth factor (VEGF) and basic fibroblast growth factor (bFGF), and inflammatory and angiogenic cytokines acting in both paracrine and autocrine manner. These activated endothelial cells acquire abnormal phenotypic and functional features (angiogenic phenotype) that may initiate and promote the development of HIV-KS [61].

Inflammatory cytokines have the capacity to reactivate latent HHV-8, resulting in an increase of HHV-8 plasma load and spread of HHV-8 in tissues, and to promote HIV replication, leading to a further production of HIV Tat protein and deterioration in host immune responses. Thus, inflammatory cytokines have the potential to perpetuate an environment conducive to HIV-KS initiation and progression [61].

## α_5_β_1 _and αVβ_3 _integrins

The interactions between cells and extracellular matrix molecules (ECM) are mediated by cell membrane receptors belonging to the integrin family, that mediate cellular migration and growth [72,73]. The increased levels of inflammatory cytokines and bFGF found in HIV-KS lesions upregulate the expression of α_5_β_1 _and αVβ_3 _integrins on HIV-KS cells [74].

In HIV-seropositive subjects, extracellular HIV Tat protein binds to α_5_β_1 _and αVβ_3 _integrin receptors on KS endothelial and spindle cells, to provide them with the necessary signals for adhesion that is required for their subsequent proliferation in response to mitogenic stimuli by bFGF. HIV Tat protein also induces the synthesis of MMP-2 and MMP-9. The increased expression of MMPs in HIV-KS cells may lead to degradation of extracellular matrix components, facilitating the locomotion of endothelial cells and invasion of spindle cells [61,74,75].

In HIV-seronegative subjects with KS, cellular growth and migration, and the expression of MMPs are induced by (α_5_β_1_)-fibronectin and (αVβ_3_)-vitronectin interactions in the presence of bFGF. The presence of HIV Tat protein in HIV-KS fortifies the α_5_β_1 _and αVβ_3 _stimulation [76,77]. This might explain the increased frequency of KS and the greater aggressiveness in clinical behaviour of HIV-KS compared to other epidemiological forms of KS.

## The role of bFGF and VEGF in the pathogenesis of HIV-KS

VEGF and bFGF are angiogenic growth factors that are powerfully expressed in HIV-KS lesions and promote angiogenesis. Both induce MMPs production by endothelial cells, and vascular permeability and subsequently oedema that is an important feature of HIV-KS [74,78]. bFGF is produced by immunoregulatory cells, that are present in HIV-KS lesions, and by activated endothelial cells [79,80]. bFGF has an instrumental role in the development of HIV-KS. It can initiate and sustain neovascularization by providing mitogenic signals to activated endothelial cells and spindle cells [32,60,81]. This concept is supported by reports that the expression of bFGF is upregulated in HIV-KS spindle cells, and antibodies to bFGF mRNA substantially reduce the angiogenic and proliferative potential of HIV-KS cells [71]. In HIV-seropositive subjects with KS, bFGF mediates Tat-induced endothelial cell proliferation, and acts with HIV Tat in promoting the production of MMPs by endothelial cells[72,82].

VEGF is a potent specific endothelial cell mitogen produced by HIV-KS endothelial and spindle cells in response to inflammatory cytokines that induce angiogenesis through autocrine mechanisms [23,83]. VEGF can synergise with bFGF to induce vascular permeability and oedema, and angiogenesis [13,23], and it is expressed and upregulated by several HHV-8 proteins including vIL-6 and vGPCR and plays a significant part in the development of KS [65].

## MMPs

MMPs are a family of proteolytic enzymes involved in degradation of extracellular matrix and basement membrane components. MMP gene expression is induced by a variety of stimuli including inflammatory cytokines, ECM-integrin interaction, growth factors, HIV Tat protein and HHV-8 proteins [13,65,75]. MMP-2 is upregulated in HIV-KS lesions and it may play a rôle in inducing vascular permeability and oedema and in promoting endothelial cell growth, angiogenesis and tumour invasion [74,84,85].

## Oxidative and nitrative metabolites in the pathogenesis of HIV-KS

Persistent inflammatory state is conducive to the production of ongoing reactive oxidative and nitrative metabolites which are associated with tumourgenesis. They promote cell proliferation, initiate nuclear and mitochondrial DNA mutations, induce a proangiogenic environment, and inactivate DNA repair enzymes [71,86].

HIV-seropositive subjects have high tissue levels of reactive oxidative and nitrative metabolites owing to increased levels of proinflammatory cytokines, more frequent opportunistic infections and a reduction in the activity of antioxidant enzymes [87]. HIV-KS lesional cells express endogenous oxidative and nitrative metabolites which together with increased exogenous oxidative and nitrative metabolite levels, associated with HIV infection, may promote the particular aggressiveness of KS in HIV-seropositive subjects [71].

## HIV infection, HAART and KS

HIV infection may directly and indirectly promote the initiation and progression of KS. HIV Tat protein, a transcriptional activator of HIV gene expression, is a major factor implicated in the pathogenesis of HIV-KS [82]. Tat protein is released by HIV-infected T cells. In this extracellular form, Tat synergises with inflammatory cytokines, which are upregulated in HIV-KS lesions, to promote angiogenesis, and progression of HIV-KS. It does this by the induction and mobilization of bFGF, and by interacting with α_5_β_1 _and αVβ_3 _integrins on both endothelial and spindle cells [75,80]. The coordinated signalling to endothelial cells integrins α_5_β_1 _and αVβ_3 _and growth factor receptors by Tat protein and bFGF respectively, are important events in the pathogenesis of HIV-KS [12,76].

By binding to α_5_β_1 _and αVβ_3 _integrins on inflammatory cytokine-activated endothelial cells, Tat protein activates the cascade of events in the FAS-ERK-MAPK intracellular signal transduction pathway. This promotes the progression of KS endothelial cells through the G1 cell cycle phase in response to bFGF stimulation, and results in increased cell proliferation [76]. In addition, Tat protein may act as an antiapoptotic agent causing prolonged survival of endothelial cells [67]. It also has the capacity to regulate the cycle of HHV-8 growth and to reactivate latent HHV-8 infection [9,13]. A further outcome of the activity of Tat protein can be an increase in the synthesis of MMP-2 by monocytes and by endothelial cells leading to increased vascular permeability and oedema [74].

HIV infection may indirectly affect the course of HIV-KS by perpetuating immunosuppression and immunodysregulation, characterized by increased production of proinflammatory cytokines that sustain the KS [13]. Moreover, Tat protein induces in monocyte-derived dendritic cells an increase in production of Th1 type cytokines and β chemokines [77].

Following the introduction of HAART sometimes established HIV-KS may regress and the likelihood of developing new lesions of KS is diminished [5,9,13,38,40]. This can be attributed to three factors. Firstly, the reduction in HIV load, Tat protein and inflammatory cytokines [13], secondly, HHV-8 specific – CD8 + cytotoxic T lymphocyte response is improved following HAART [13,88,89] and therefore there is a reduction in HHV-8 load. Thirdly, some protease inhibitors that are administered as a component of HAART have anti-inflammatory and anti-angiogenic activity, thus directly inhibiting HIV-KS [10].

However, despite otherwise effective HAART, established HIV-KS may not always regress, and new KS lesions may appear [13,54], perhaps as a manifestation of immune reconstitution inflammatory syndrome (IRIS) [30,33,55,56,59].

## HIV associated IRIS

IRIS can be defined as an exuberant immune-mediated inflammatory response to a pre-existing subclinical pathogen or tumour antigen after treatment has brought about an improvement in a host previously profoundly depressed immunity [55,59,90]. In the context of HIV infection, HAART-induced IRIS has been described in relation to opportunistic infections including herpes simplex (HS), herpes zoster (HZ), mycobacterium tuberculosis, in relation to autoimmune thyroid disease, and in relation to KS. HIV-IRIS occurs paradoxically despite a reduction in HIV load and improvement in all HIV related immunologic parameters early after the introduction of HAART, and is probably the result of reconstituted pathogen-specific immune responses [33,89,91-96]. However, the details regarding the immunopathogenic mechanisms that bring about IRIS are speculative [96].

In HIV-seropositive subjects who start HAART at an early stage of HIV infection, the number and function of CD4+ T cells tend to return to normal. On the other hand, subjects who start HAART, when the HIV infection is moderately advanced (CD4+ T cell counts are between 100 × 10^6^/L and 300 × 10^6^/L), will not show a similar recovery. However, even such a partial immune reconstitution results in a profound decline in HIV-associated morbidity and mortality [97].

CD4+ T cell restoration in peripheral blood after HAART is biphasic. An initial rapid increase during the first 12 weeks of treatment is followed by a more gradual increase over the remainder of the first year, and after that there is usually no further significant improvement. The initial- phase increase in the CD4+ T cells is due to proliferation and reduced apoptosis of existing memory cells in the circulation and to redistribution of CD4+ T cells from the lymph nodes into the circulation. Only after several months of HAART, will T lymphoiesis associated with improvement of thymic function become evident, giving rise to increased numbers of naïve T lymphocytes [89,97-105]. With continued HAART there will also be an increase in CD8+ T cells in the circulation lagging behind the peak increase in CD4+ T cells by a period of about 5 weeks. During the initial immune reconstitution phase the ability of the host to mount immuno-inflammatory responses is restored [33,106], owing to the partial recovery of CD4+ T cells and CD8+ cytotoxic T cell responses, and the shift from a Th-2- to a Th-1-dominant cytokine profile.

All HIV-IRIS events occur in subjects who display as indicators of immune reconstitution, a decrease in HIV viral load and an increase in CD4+ T cell count. HIV-seropositive subjects with IRIS episodes tend to be younger at the time of introduction of HAART and tend to have a lower median baseline CD4+ T cell percentage than HIV-seropositive subjects who do not experience IRIS. Overall, the median time to onset of IRIS in those subjects who display this response is 12 weeks [92-96].

HIV-seropositive subjects who are at greater risk for developing IRIS are those with low CD4+ T cell counts of < 100 cells/ul [95,107], those with CD4+ T cell percentage of <10%, and those of a younger age at the time of introduction of HAART [96]. There is no association between the risk for developing IRIS and the magnitude of the increase in CD4+ T cell count or the percentage of CD4+ T cell increase, and the plasma load decrease following HAART [95,96,108] In contrast, Shelburne et al.[94] and Breton et al.[109] found that HIV-IRIS is associated with a greater increase in the percentage of CD4+ T cell one month after the introduction of HAART and with a more pronounced and persistent reduction in HIV load. Thus, there is some conflict in the immunological parameters associated with the development of HIV-IRIS.

The definite association of the events of HIV-IRIS with a low baseline CD4+ T cell percentage probably reflects a higher burden of opportunistic subclinical pathogens at the time of HAART introduction. Such a high burden of antigenic stimulation together with a dysregulated increased immune response during immunoreconstitution after HAART, may be responsible for the development of HIV-IRIS [90].

The immunopathogenic mechanisms associated with IRIS differ according to the type of pathogen involved [90]. CD8+ cytotoxic T cell response is associated with IRIS induced by viral infections [110,111] and a delayed type- hypersensitivity or a lymphoproliferative reaction with IRIS induced by mycobacterial infections [90,112,113].

## IRIS associated HIV-KS

Most HIV-seropositive subjects with KS do not demonstrate HHV-8-specific cytotoxic T lymphocyte response. This lack of HHV-8-specific cellural immune response during HIV infection is a contributory factor in the development of HIV-KS. The decline in the incidence of HIV-KS, and the regression of KS in some HIV-seropositive subjects after the introduction of HAART, suggests that some general improvement in immunity and in the recovery of HHV-8-specific, MHC class I-restricted cytotoxic CD8+ T cell response could be important in the control of HHV-8 replication [114,115]. The CD4+ and CD8+ T cells that increase shortly after the introduction of HAART are memory cells. It is probable that in HIV-seropositive subjects who are commonly latently or subclinically coinfected with HHV-8, the HHV-8-specific cytotoxic T cells lodge in sites of HHV-8 subclinical infection, or in sites of established KS lesions. In most of HIV-seropositive subjects, these CD8+ T cells will be protective and will control HHV-8 replication and spread, thus reducing the incidence of new KS lesions, and at times bringing about regression in established KS lesions. However in a minority of HIV-seropositive subjects with subclinical HHV-8 infection, or with established KS, such an immune response may be dysregulated and accompanied by an intensified HHV-8-specific inflammatory response paradoxically causing a worsening of KS and as a consequence, the development of IRIS-KS [112].

There could be other mechanisms that may be involved in the pathogenesis of IRIS-KS. As part of the immune restoration after HAART, there is a shift in the cytokine profile from the Th-2 to the Th-1 type. Th-1 cytokines have the capacity to reactivate latent HHV-8 in blood and tissue cells, and as a consequence, HHV-8 can spread to uninfected endothelial cells. Perhaps in a subset of HIV-seropositive subjects, the restored HHV-8-specific immune response following HAART is ineffective in controlling the increased HHV-8 antigens in blood and tissue cells, and as a result, paradoxically promotes the development of IRIS-KS.

Because of the inability of the immune response to control the HHV-8 infection, two processes may occur in parallel; the development of an exaggerated immunoinflammatory reaction characterized by the increased production of inflammatory cytokines in order to combat HHV-8; and secondly, the increase in the HHV-8 load in the tissues through autocrine and paracrine mechanisms increase the production of inflammatory cytokines, chemokines and growth factors. Together these processes upregulate the expression of αvβ_3 _and α_5_β_1_, and MMPs that induce the IRIS-KS angioproliferation and tumourigenesis [55]. In this context, HIV Tat protein and a profound state of immunosuppression do not significantly affect the development of IRIS-KS.

Bower et al. [33], reported that 10 of 150 (6.6%) HAART-naïve HIV-seropositive subjects with KS either developed new KS lesions or exhibited rapid progression of existing KS lesions within 2 months of starting HAART. These subjects had a significantly higher CD4+ T cell count at the time of IRIS-KS diagnosis and a higher frequency of KS-associated oedema than HIV-seropositive subjects with KS who did not develop IRIS.

## Summary

The clinical course of HIV-KS is unpredictable. For some HIV-seropositive subjects, KS is a mild disease, while for others it may be rapidly progressive and aggressive. Regression of HIV-KS lesions and a substantial decrease in the incidence of HIV-KS occur after the introduction of HAART. However, a small subset of HIV-seropositive subjects with a low CD4 + T cell count at the time of HAART initiation, may develop IRIS associated HIV-KS shortly thereafter.

IRIS-KS is not a specific entity but it is a phenomenon which can be recognized by certain well defined circumstances: the presence of low CD4+ T cell counts at the time of introduction of HAART; a temporal relationship between rapid clinical progression of KS and the development of new KS lesions usually of about 8 weeks (though this period may range from 3 weeks to 22 weeks) after the initiation of HAART; and the suppression of HIV load and restoration of CD4+ T cell count.

## Competing interests

The author(s) declare that they have no competing interests.
